# Predictive modeling of skin permeability for molecules: Investigating FDA-approved drug permeability with various AI algorithms

**DOI:** 10.1371/journal.pdig.0000483

**Published:** 2024-04-03

**Authors:** Rami M. Abdallah, Hisham E. Hasan, Ahmad Hammad

**Affiliations:** 1 Department of Pharmaceutical Sciences, Faculty of Pharmacy, Zarqa University, Zarqa, Jordan; 2 Department of Artificial Intelligence, Faculty of Information Technology, Middle East University, Amman, Jordan; Amity University - Mumbai Campus, INDIA

## Abstract

The transdermal route of drug administration has gained popularity for its convenience and bypassing the first-pass metabolism. Accurate skin permeability prediction is crucial for successful transdermal drug delivery (TDD). In this study, we address this critical need to enhance TDD. A dataset comprising 441 records for 140 molecules with diverse LogK_p_ values was characterized. The descriptor calculation yielded 145 relevant descriptors. Machine learning models, including MLR, RF, XGBoost, CatBoost, LGBM, and ANN, were employed for regression analysis. Notably, LGBM, XGBoost, and gradient boosting models outperformed others, demonstrating superior predictive accuracy. Key descriptors influencing skin permeability, such as hydrophobicity, hydrogen bond donors, hydrogen bond acceptors, and topological polar surface area, were identified and visualized. Cluster analysis applied to the FDA-approved drug dataset (2326 compounds) revealed four distinct clusters with significant differences in molecular characteristics. Predicted LogK_p_ values for these clusters offered insights into the permeability variations among FDA-approved drugs. Furthermore, an investigation into skin permeability patterns across 83 classes of FDA-approved drugs based on the ATC code showcased significant differences, providing valuable information for drug development strategies. The study underscores the importance of accurate skin permeability prediction for TDD, emphasizing the superior performance of nonlinear machine learning models. The identified key descriptors and clusters contribute to a nuanced understanding of permeability characteristics among FDA-approved drugs. These findings offer actionable insights for drug design, formulation, and prioritization of molecules with optimum properties, potentially reducing reliance on costly experimental testing. Future research directions include offering promising applications in pharmaceutical research and formulation within the burgeoning field of computer-aided drug design.

## Author summary

Our study delves into the exciting realm of transdermal drug delivery, a growing preference for patients due to its convenience. Recognizing the challenge posed by the skin’s natural barrier to drug permeation, we employed advanced machine learning models to predict skin permeability solely based on descriptors computationally calculated from the chemical structure of the molecule. Key descriptors, including partition coefficient, hydrogen bond donors, hydrogen bond acceptors, and topological polar surface area, emerged as influential factors in skin permeability prediction. Hydrophobicity, polarity, and adherence to Lipinski rules were identified as crucial considerations. Analyzing FDA-approved drugs and their predicted permeability revealed distinct clusters with varied permeability characteristics, shedding light on the potential applications of different compounds in transdermal drug delivery. Our research provides valuable tools for early-stage drug discovery, facilitating the selection of compounds with optimal skin permeability. While acknowledging certain limitations, particularly in representing high molecular weight drugs, our models offer a promising avenue for efficient drug design and formulation. By making these findings accessible, we aim to contribute to the broader understanding of transdermal drug delivery and inspire further research in this dynamic field.

## Introduction

Recently, there has been increasing interest in utilizing the skin as a convenient route for drug administration, both for local and systemic therapeutic effects [1,2]. However, several challenges are present for the effective delivery of drugs through the skin, which forms a natural barrier for the permeation of xenobiotics, and the development of complicated pharmaceutical technology (i.e., transdermal drug delivery, which is of interest) is even more challenging [3]. The most notable one of them is that the drug must have suitable physicochemical properties to enable it to penetrate the stratum corneum and reach the bloodstream with a sufficient dose [4]. Currently, formulation scientists rely on empirical rules to select drugs for transdermal drug delivery (TDD), but many drugs deviate from these rules and exhibit varied behavior [5,6]. Moreover, these rules provide a qualitative estimation of permeability, and it is generally difficult to compare drugs that obey these rules. Therefore, with the accumulated data on the permeability of drugs, it is essential to develop appropriate models that provide accurate quantitative predictions for the skin permeability of drugs [7].

*In silico* QSPR (Quantitative Structure-Property Relationship) models, which correlate numerical descriptors of molecular structure with specific properties, have been extensively used to predict skin permeability [8]. Multiple linear regression (MLR) and principal component analysis (PCA) models have traditionally been employed in QSPR studies of skin permeability [9,10]. However, due to the complex and diverse nature of the chemicals involved, nonlinear regression methods such as support vector machine (SVM), random forest (RF), and artificial neural networks (ANN) have gained popularity over linear methods. These nonlinear methods are more effective at identifying patterns and capturing nonlinear relationships within complex datasets [7,8].

Artificial intelligence (AI) is a rapidly evolving field that aims to design and build machines capable of performing tasks requiring human intelligence, such as problem-solving, learning, and decision-making. AI has immense potential to improve various domains, including healthcare and drug development [11]. In the context of drug delivery, AI can be utilized to develop models for predicting drug permeability and bioavailability based on their physicochemical properties. This has the potential to accelerate the drug development process and identify promising drug candidates that may have been overlooked [12]. Several AI models have been developed to estimate skin permeability using basic physicochemical characteristics [7,13–18]. Most of these models rely on calculated descriptors for model training in order to determine the permeability coefficient (k_p_). More sophisticated models, such as ANN, have started to be commonly employed for modeling and predicting the properties and behavior of molecules by simulating the learning and generalization behavior of the human brain for complex multidimensional problems [19].

The objective of this study is to develop a regression model utilizing various AI algorithms, including nonlinear models, for predicting LogK_p_ of new compounds based solely on their molecular structure. The proposed model will be utilized for the prediction of LogK_p_ values for FDA-approved drugs. Subsequently, cluster analysis will be employed to categorize these drugs into distinct classes based on their descriptors in order to elucidate their permeability patterns across a diverse range of molecular structures. Additionally, an investigation of the drug permeability patterns will be conducted through their classification according to the Anatomical Therapeutic Chemical (ATC) code.

## Methods

### Study design

**Fig 1:**
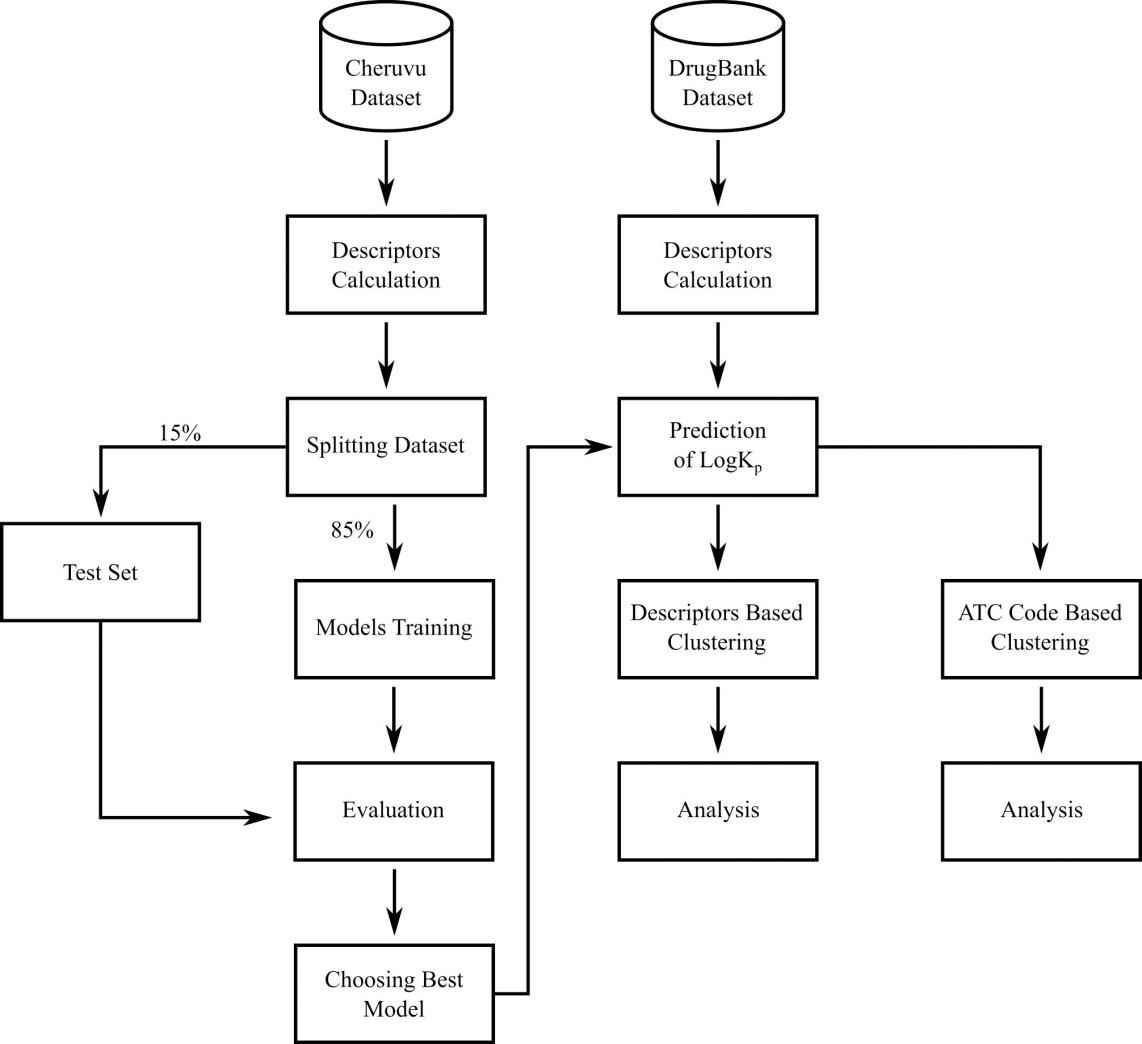
Workflow of Predictive Model Development and FDA-Approved Drug Classification.

This research aims to develop a predictive model utilizing AI algorithms to predict skin permeability and classify FDA-approved drugs based on their physicochemical properties and skin permeability patterns. Fig 1 provides a visual representation of the systematic study design workflow, leveraging computational approaches and machine learning techniques. The selected dataset served as the basis for both training and evaluating the regression predictive models. It underwent partitioning into an 85% training set and a 15% test set. Subsequently, the models were trained and evaluated, followed by the selection of the best-performing model for subsequent analysis. In the exploration of the permeability patterns of FDA-approved drugs, a DrugBank dataset was used, the descriptors were calculated, a prediction of skin permeability was made, and lastly, a clustering process was conducted.

The rationale behind this descriptor-based clustering lies in its potential applications in facilitating the selection of drug candidates for transdermal formulation, aligning with industry standards such as the Biopharmaceutical Classification System (BCS), which categorizes drugs based on their water solubility and intestinal permeability into four categories (I to IV) [20]. The classification of drugs into distinct classes enables a systematic understanding of drug behavior. Furthermore, FDA-approved drugs were grouped according to the first two levels of the ATC code, which categorize them based on their therapeutic uses and pharmacological classes, and a comparative analysis of permeability was conducted across them. This demonstrates how different pharmacologic and therapeutic groups have different distributions of skin permeability.

The study involved the analysis of publicly available data, and ethical considerations primarily revolved around ensuring the responsible and accurate use of the data. No human or animal subjects were involved in the study. The code used for analysis is available in the GitHub public repository (https://github.com/AhmadHammad21/Skin-Permeation).

### Skin pereambility dataset

In this study, a skin permeability dataset was acquired from the work of Cheruvu *et al*. [21]. The dataset encompasses *in vitro* human skin permeation parameters, including LogK_p_ values, for a diverse range of molecules, including drugs, xenobiotics, and other chemical compounds. It provides essential information on physicochemical properties, experimental conditions, and solute behavior on human epidermal membranes. The stringent inclusion criteria applied in the assembly of the dataset, which encompassed considerations such as limiting the membranes to human epidermal and isolated stratum corneum, exclusive inclusion of reports involving undamaged skin and corresponding skin integrity tests, incorporation solely of data derived from aqueous solutions and buffers as a vehicle, and the selection of unionized solutes with a fraction unionized (*f*_*ui*_) exceeding 0.9, served as a valuable and updated resource to utilize in developing a robust skin permeability prediction model. In order to maintain a homogeneous dataset, water and permanently ionized molecules were excluded. We then retrieved the SMILES structures of the compounds from PubChem for generating descriptors in subsequent analyses.

### Calculation of molecular descriptors

To generate descriptors for the molecules, we utilized the open-source, Java-based chemoinformatics library Chemistry Development Kit (CDK) version 2.8 [22]. The SMILES structure of the molecules was imported into the library, and a comprehensive set of one-dimensional and two-dimensional representations of molecular structures (1D/2D) descriptors were calculated for subsequent use as inputs in the AI models. These descriptors take into account the 1D molecular chemical formula and the 2D spatial and topological information of the structure when calculating the descriptor value. Prior to the descriptor calculation, salts were neutralized. To handle missing data, we filled the columns containing errors with the mean or median value of the respective column.

### AI models

Two types of experiments were conducted, namely regression and cluster analysis. The experiments were carried out using Scikit-Learn version 1.2 [23], which is an open-source library utilizing the Python programming language. The process involved various stages, including data cleaning, preprocessing, and splitting into training and testing sets. To ensure fair and effective model training, the molecular descriptors were subjected to a standardization process. Regarding regression models, the dataset was split into an 85% training set and a 15% testing set. Subsequently, the model with optimal performance was selected for further analysis.

### Regression models

To predict the LogK_p_ values, we constructed a diverse set of models using the Scikit-Learn library. These models encompassed various algorithms, including MLR and ensemble methods such as bagging and boosting. These methods leverage the capabilities of multiple machine learning models by integrating their predictions, thereby surpassing the performance of individual models [24]. Two prominent techniques are employed for model combinations. Bagging is where models are independently trained and their outputs are combined at the end. Conversely, boosting occurs when each successive model learns from the predictive shortcomings of the previous model. The MLR model served as the baseline approach, leveraging the relationship between the input descriptors and LogK_p_ values for predictions.

Ensemble algorithms such as RF, XGBoost, CatBoost, and LGBM were employed. Additionally, we utilized ANNs, an advanced machine learning model, to capture complex relationships within the descriptors and enhance the predictive performance of the models. The models were trained using a comprehensive dataset of molecule descriptors, enabling them to learn the underlying patterns and relationships that govern permeability behavior.

### Model evaluation and validation

To evaluate the performance and accuracy of the developed models, we employed several evaluation metrics to compare the predicted LogK_p_ values with the actual values in the test set. These metrics included R-Squared (R^2^)^(1)^, Root Mean Square Error (RMSE)^(2)^, and Mean Absolute Error (MAE)^(3)^. R^2^ serves as an indicator of the degree to which the regression model aligns with the actual values. RMSE quantifies the root average of the squared difference between the predicted and actual LogK_p_ values, while MAE provides a measure of the average absolute difference. The model performance on the training set was evaluated using a 5-fold cross-validation MAE.


$$R^{2}=1-\frac{\sum_{i=1}^{n}{\left(y_{i}-{\hat{y}}_{i}\right)}^{2}}{\sum_{i=1}^{n}{\left(y_{i}-\bar{y}\right)}^{2}}$$ (1)

$$RMSE=\sqrt{\frac{1}{n}\sum_{i=1}^{n}{\left(y_{i}-{\hat{y}}_{i}\right)}^{2}}$$ (2)

$$MAE=\frac{1}{n}\sum_{i=1}^{n}\left|y_{i}-{\hat{y}}_{i}\right|$$ (3)

### Cluster analysis

For investigating the permeability of approved drugs, we obtained a dataset of FDA-approved drugs from DrugBank, which is a comprehensive online database that provides information on drugs, drug targets, and drug interactions [25]. Preprocessing steps were applied to ensure data integrity, including the removal of salts, the exclusion of inorganic drugs, and drugs with multiple bioactive compounds. We predicted the permeability of these approved drugs at 37°C and conducted cluster analysis using K-means clustering, an unsupervised learning approach, and a data mining technique that identifies similarities between data points. The number of clusters was chosen based on the results of the elbow method. Furthermore, we utilized the ATC code for each drug to classify them based on the first two levels of the code. The ATC code is a widely used classification system that categorizes drugs based on their therapeutic properties and anatomical targets [26]. It provides a hierarchical structure with five levels, where the first level represents the main anatomical group and the second level represents the therapeutic subgroup. Groups with less than three drugs were excluded from the statistical analysis.

### Statistical analysis

Statistical analysis was performed using PSPP 2.0.0 (GNU Project). Group comparisons were conducted using the Kruskal-Wallis test, and pairwise comparisons were conducted using the Mann-Whitney U test. A significance threshold of *p* < 0.05 was applied to determine significant differences between groups.

## Results

### Characterization of the dataset

**Fig 2:**
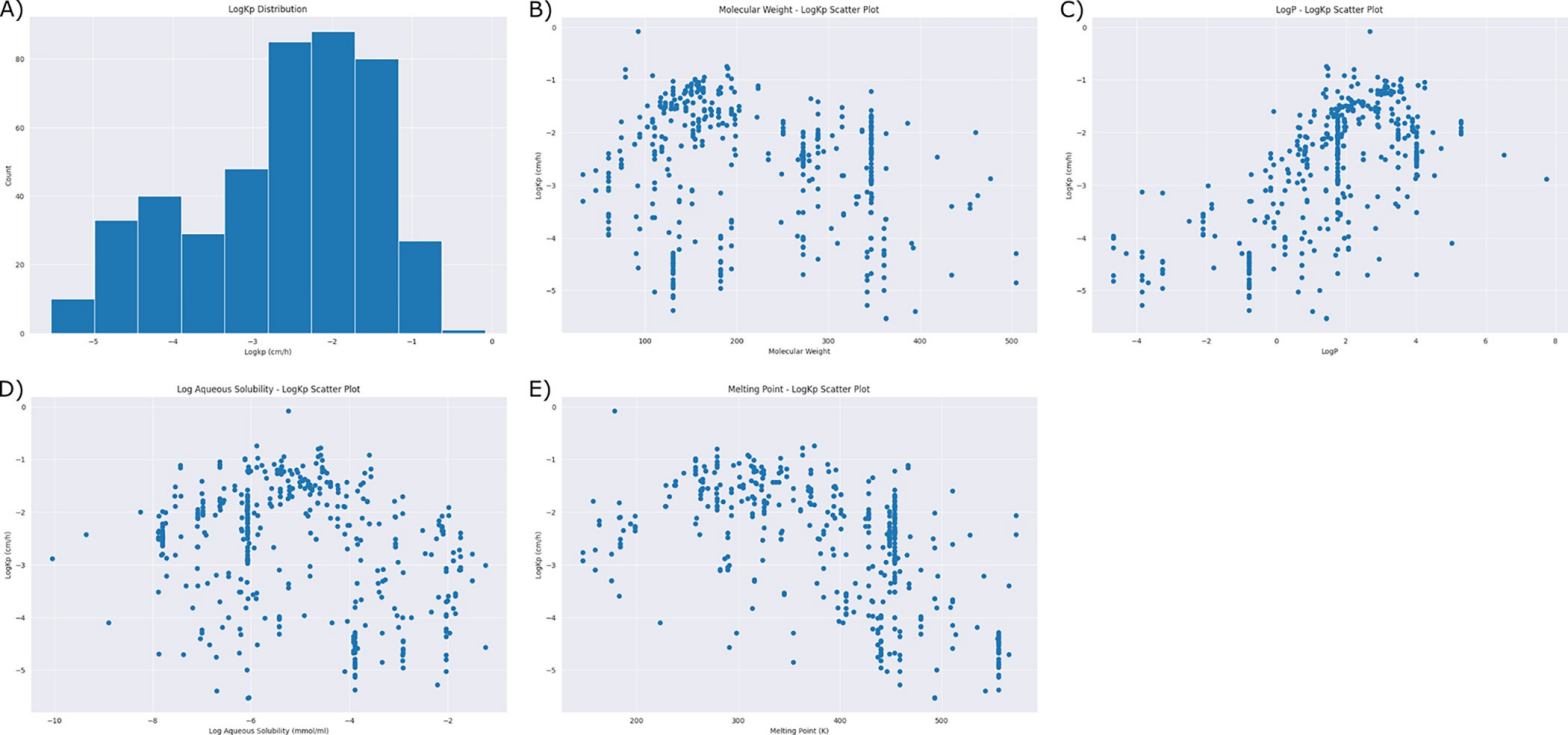
Comprehensive Characterization of the Dataset for 140 Molecules. The distribution of the molecules according to: **(A)** LogK_p_. The distribution of LogK_p_ values is depicted alongside with **(B)** molecular weight, **(C)** LogP**, (D)** Log aqueous solubility, **(E)** melting point.

The acquired dataset consists of 441 records for 140 different molecules with a diverse range of LogK_p_ values. The values ranged from -5.53 cm/h to -0.08 cm/h and were measured under varying temperatures, ranging from 295 K to 312 K. Fig 2 provides a comprehensive characterization of the dataset, including the distribution of LogK_p_ values, molecular weight, octanol water partition coefficient (LogP), water solubility, and melting point. Specifically, the figure illustrates (a) the distribution of LogK_p_ values, while (b), (c), (d), and (e) exhibit the distribution of molecular weight, LogP, water solubility, and melting point, respectively. This figure offers insights into the dataset’s variability and suitability for further analysis and regression model development.

### Descriptors calculation

**Fig 3:**
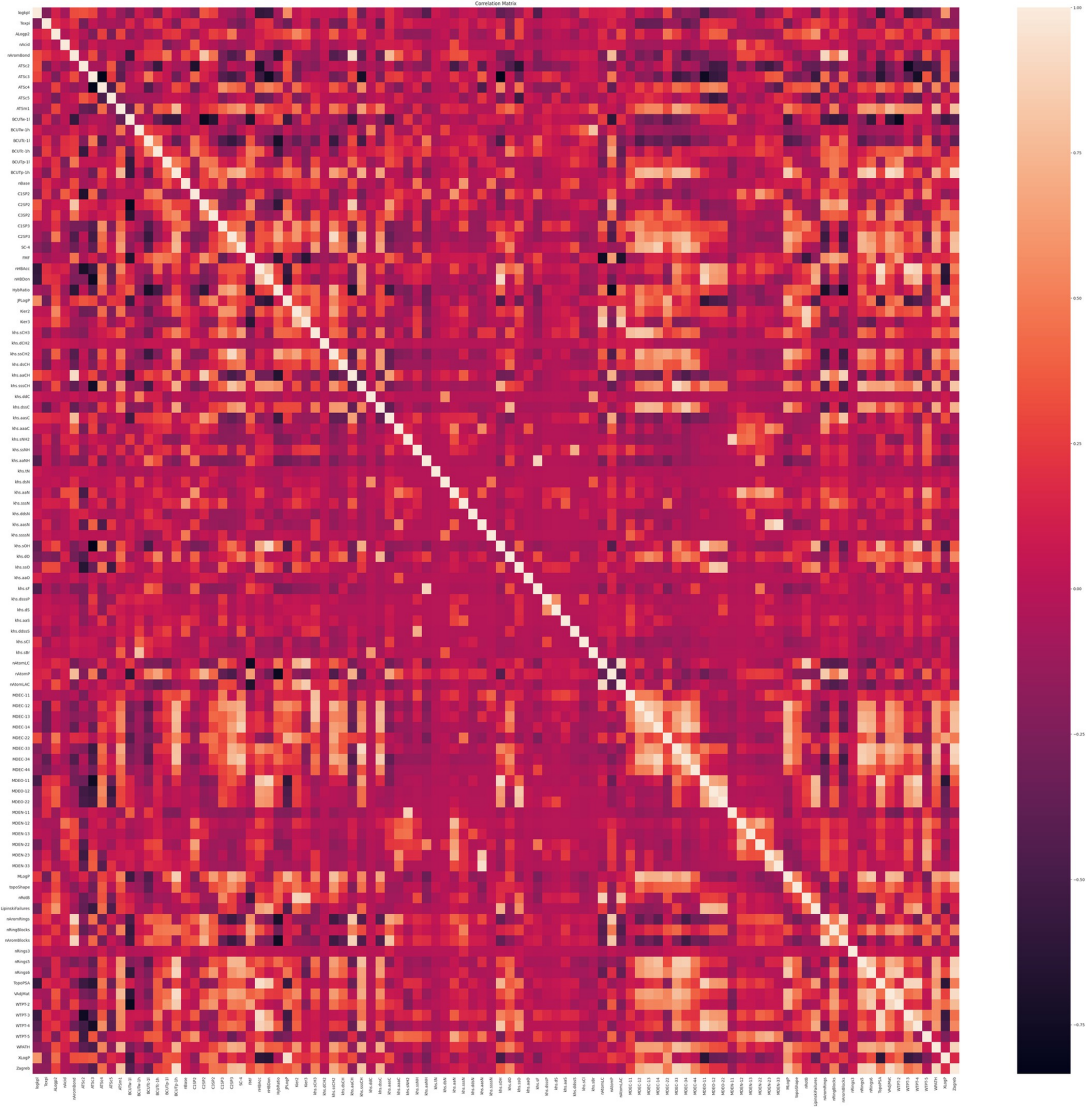
Correlation Heatmap of Different Molecular Descriptors for Skin Permeability.

**Fig 4:**
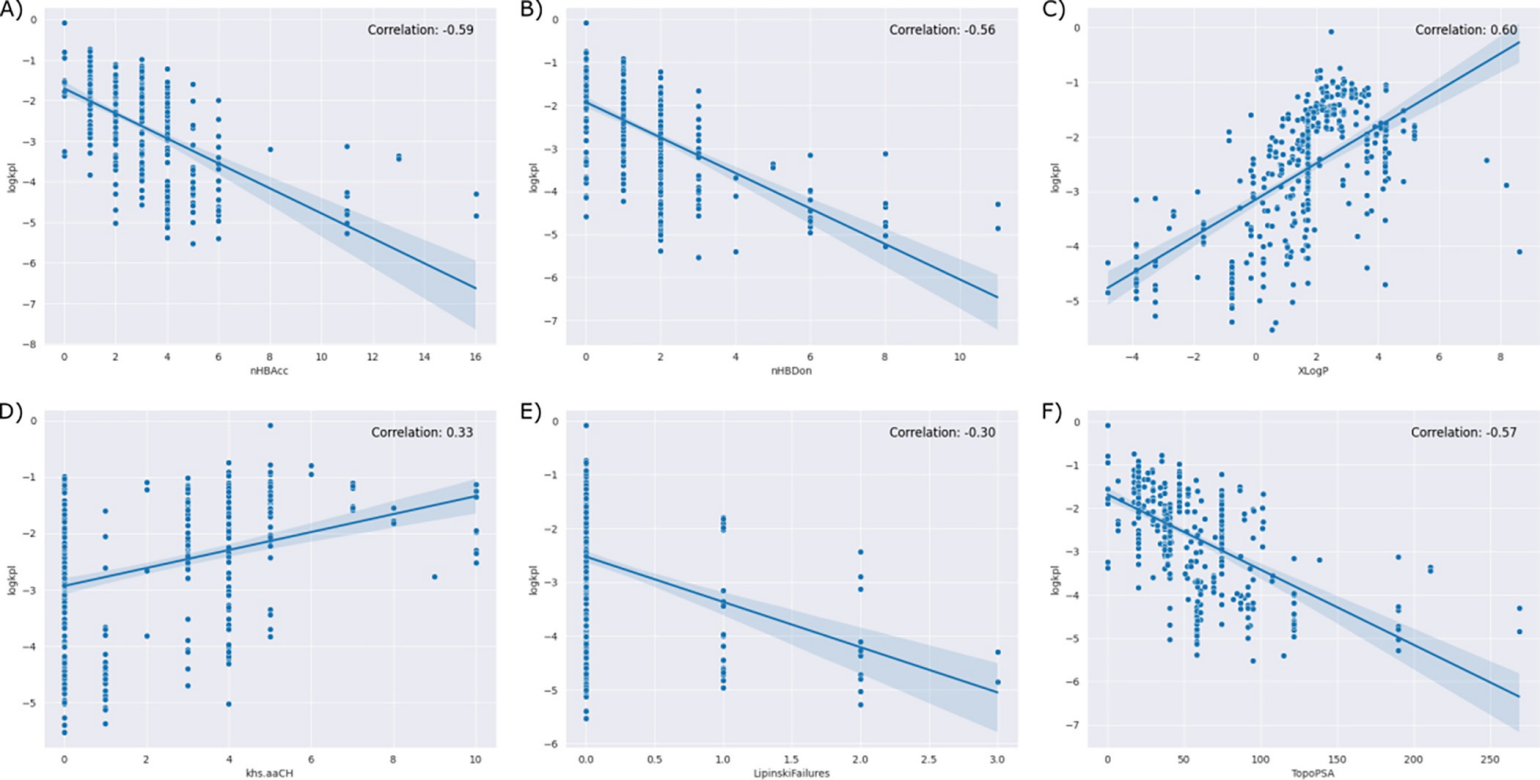
Correlation Analysis of Key Molecular Descriptors Influencing Skin Permeability (LogK_p_). This figure highlights the relationship between LogK_p_ and specific descriptors crucial for skin permeability. Panels (A) through (F) showcase highly correlated descriptors: **(A)** number of hydrogen bond acceptors, **(B)** number of hydrogen bond doners, **(C)** calculated LogP (XLogP), **(D)** number of aromatic CH carbons, **(E)** number of Lipinski rule failures, and **(F)** topological polar surface area.

A total of 222 (1D/2D) descriptors were computed for the compounds present in the dataset. Descriptors with zero values and highly correlated descriptors with absolute correlation factor of 0.95 and higher were excluded from further analysis, ending with 145 descriptors. Fig 3 shows a heatmap depicting the correlations between different molecular descriptors. As expected, there is a strong negative correlation between LogK_p_ values and descriptors such as polar surface area, number of hydrogen bond donors, and number of hydrogen bond acceptors. On the other hand, LogK_p_ shows a strong positive correlation with LogP descriptors, indicating that the permeability of molecules through the skin is significantly influenced by their hydrophobic nature. The number of Lipinski empirical rule failures also shows a negative correlation with LogK_p_, suggesting that the Lipinski rules can be extended to predict skin permeability. Notably, carbohydrates exhibit the highest number of Lipinski rule failures with very low LogK_p_ values (-4.53 ± 0.62 cm/h). Fig 4 illustrates the correlation of a subset of highly correlated descriptors with LogK_p_.

### Regression models development

Various machine learning models, including MLR, RF, XGBoost, CatBoost, LGBM, and ANN, were employed for regression analysis. Table 1 presents the performance of these models based on metrics such as RMSE, MAE, and R^2^ score.

**Table 1:** Performance of various machine learning models.

Model	R^2^	RMSE	MAE	Cross Validation MAE
**MLR (10 features)**	0.338	0.814	0.624	0.599
**Decision Tree**	0.729	0.535	0.323	0.473
**RF**	0.788	0.473	0.314	0.444
**XGBoost**	0.798	0.462	0.281	0.446
**Gradient Boosting**	0.818	0.439	0.276	0.441
**CatBoost**	0.797	0.464	0.300	0.436
**LGBM**	0.819	0.437	0.278	0.445
**ANN**	0.797	0.462	0.298	0.412

**Fig 5:**
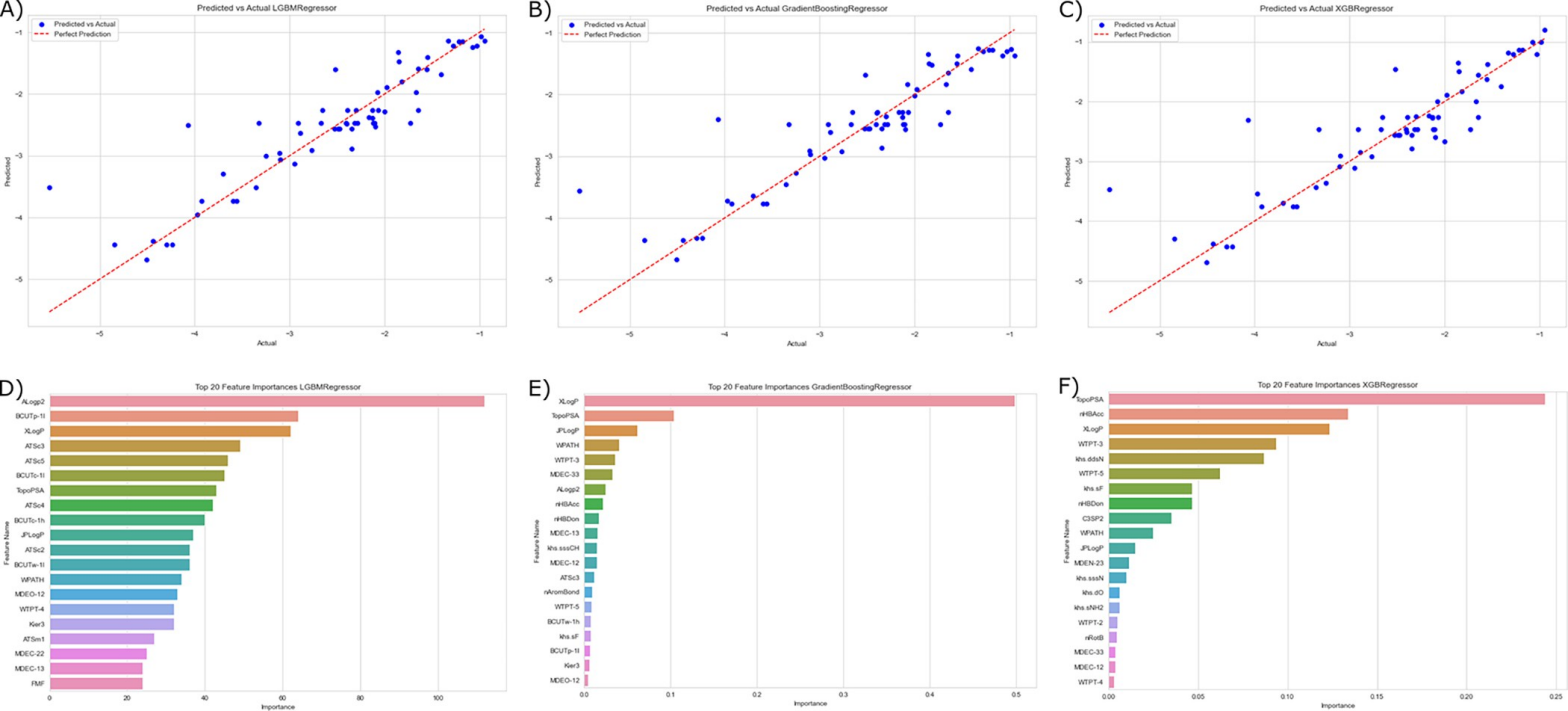
The Predicted versus Actual Plot of the Best Three Machine Learning Models for Predicting LogK_p_ Along with Their Top-Performing Features.

Based on the reported metrics, the LGBM, XGBoost, and Gradient Boosting models outperformed the other models in terms of MAE, RMSE, and R^2^. Among these models, LGBM exhibited the lowest RMSE along with the highest R^2^ value, indicating its superior performance and ability to accurately predict LogK_p_ values, while gradient boosting showed the lowest MAE. The ANN model also demonstrated a performance slightly lower than most of the ensemble boosting models. Conversely, the MLR model exhibited the lowest performance when utilizing all descriptors. Since MLR typically achieves better results with a smaller number of descriptors, we applied forward feature selection to identify the most relevant 10 descriptors and reconstructed the model. However, even with this reduced set of descriptors, MLR still yielded the highest MAE, RMSE, and the lowest R^2^ value among the models assessed. Fig 5 shows the predicted compared to actual values of LogK_p_ along with the top important features for the best three machine learning models, namely LGBM, gradient boosting, and XGBoost. Polarity descriptors were among the most important features in predicting LogK_p_ values. For further analysis, the LGBM model was used for predicting LogK_p_.

### Cluster analysis

**Fig 6:**
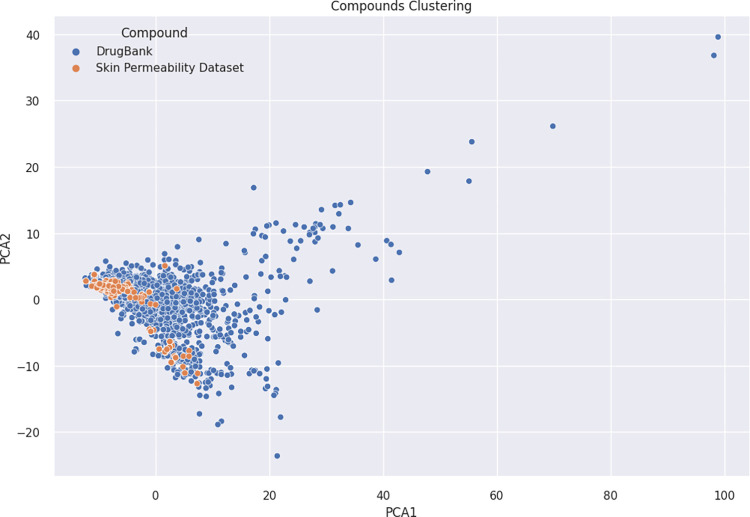
Overlay of Skin Permeability Dataset onto DrugBank FDA-Approved Drugs Using PCA. The overlay visualizes the distribution of skin permeability dataset across the FDA-approved drug compounds from the DrugBank dataset.

**Fig 7:**
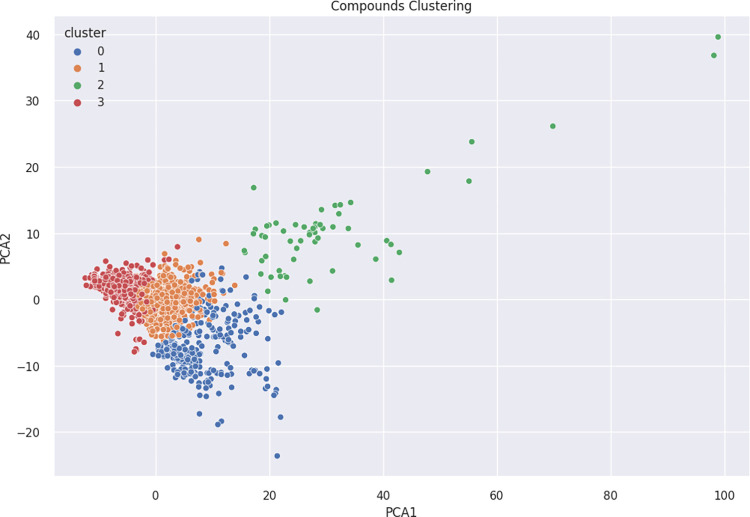
Cluster Analysis of FDA-Approved Drugs from the DrugBank Dataset.

**Fig 8:**
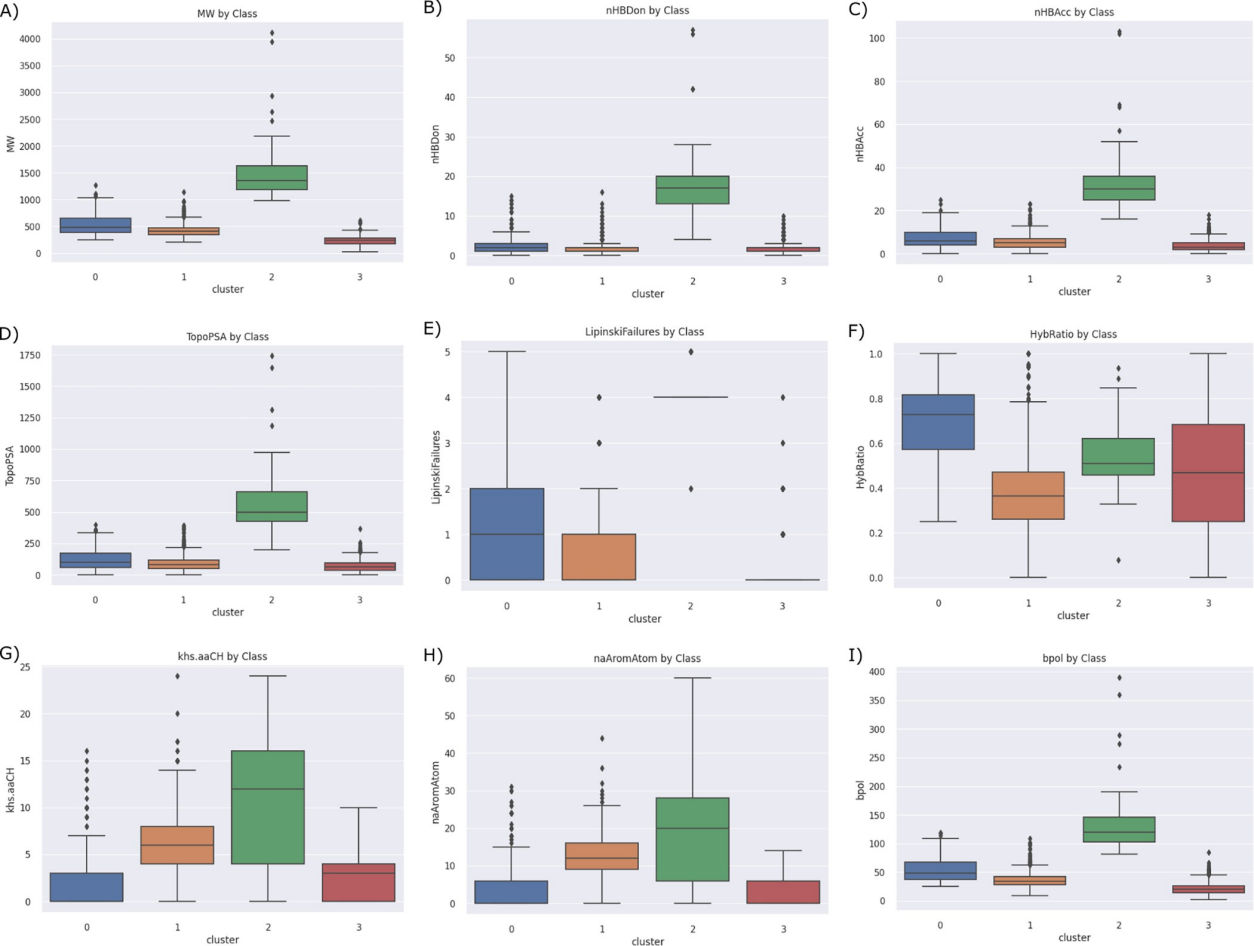
Descriptive Analysis of Clustered FDA-Approved Drugs from the DrugBank Dataset. Box plots display selected molecular descriptors for the identified clusters, illustrating distinct properties among the clusters. (A) molecular weight, *p* < 0.0001 between all, (B) number of hydrogen bond doners, *p* < 0.0001, except between 1 and 3, *p* = 0.094, (C) number of hydrogen bond acceptors, *p* < 0.0001, (D) topological polar surface area, p < 0.0001 (E) number of Lipinski failures, *p* < 0.0001, (F) hybridization ratio (ratio of Sp^3^ carbons to the total of Sp^3^+Sp^2^ carbons), *p* < 0.0001, except between 2 and 3, *p* = 0.027, (G) number of aromatic carbons, *p* < 0.0001, (H) number of aromatic atoms, *p* < 0.0001, and (I) total absolute sum of polarizability difference between bonded atoms, *p* < 0.0001.

The DrugBank dataset of FDA-approved drugs (2326 compounds) underwent descriptor calculation followed by PCA to extract two principal components, which collectively accounted for 43% of the total variance in the dataset. In Fig 6, the overlay of the skin permeability dataset onto the DrugBank dataset was achieved by applying the same PCA algorithm used for the DrugBank dataset, highlighting areas of high density and variability in covering the diverse FDA-approved drug compounds. A cluster analysis was conducted using the K-means algorithm, resulting in four distinct clusters shown in Fig 7. While the clusters did not exhibit a clear demarcation, these clusters exhibited significantly different properties, as depicted in Fig 8. Descriptive statistics for these properties are provided in S1 Table. Among the identified clusters, a Kruskal-Wallis test for molecular weight showed that there was a statistically significant difference between groups (*p* < 0.0001). Class 2 exhibited the highest average molecular weight (1529.25 ± 605.62 Daltons). This class predominantly comprises polypeptides. Following Class 2, Class 0 had an average molecular weight of 540.98 ± 193.56 Daltons, while Class 1 had an average molecular weight of 423.85 ± 116.12 Daltons. On the other hand, Class 3 encompassed very small molecules with an average molecular weight of 231.17 ± 78.60 Daltons. Furthermore, an analysis of the number of hydrogen bond donors and acceptors revealed that Class 2 had statistically significant higher values in this regard compared to the others (*p* < 0.0001). This observation aligns with the fact that Class 2 primarily consisted of polypeptides, which tend to exhibit a higher number of hydrogen bonding sites. Additionally, Class 2 compounds exhibited the highest topological polar surface area (*p* < 0.0001), indicating their pronounced polarity.

The analysis also revealed additional insights into their chemical composition. Specifically, Class 0 was found to have a higher proportion of saturated carbons compared to Class 1 (*p* < 0.0001), which exhibited a greater number of aromatic atoms (*p* < 0.0001). This disparity in carbon saturation suggests a variation in the structural characteristics of the compounds between these two classes. Moreover, Class 0 displayed a higher occurrence of Lipinski rule violations compared to Class 1 (*p* < 0.0001). This observation can be attributed to the relatively higher molecular weight and number of hydrogen bond donors and/ or acceptors present in Class 0 compounds. Furthermore, Class 2 compounds predominantly exhibited four Lipinski failures. In contrast, Class 3 compounds had predominantly zero Lipinski failures.

**Fig 9:**
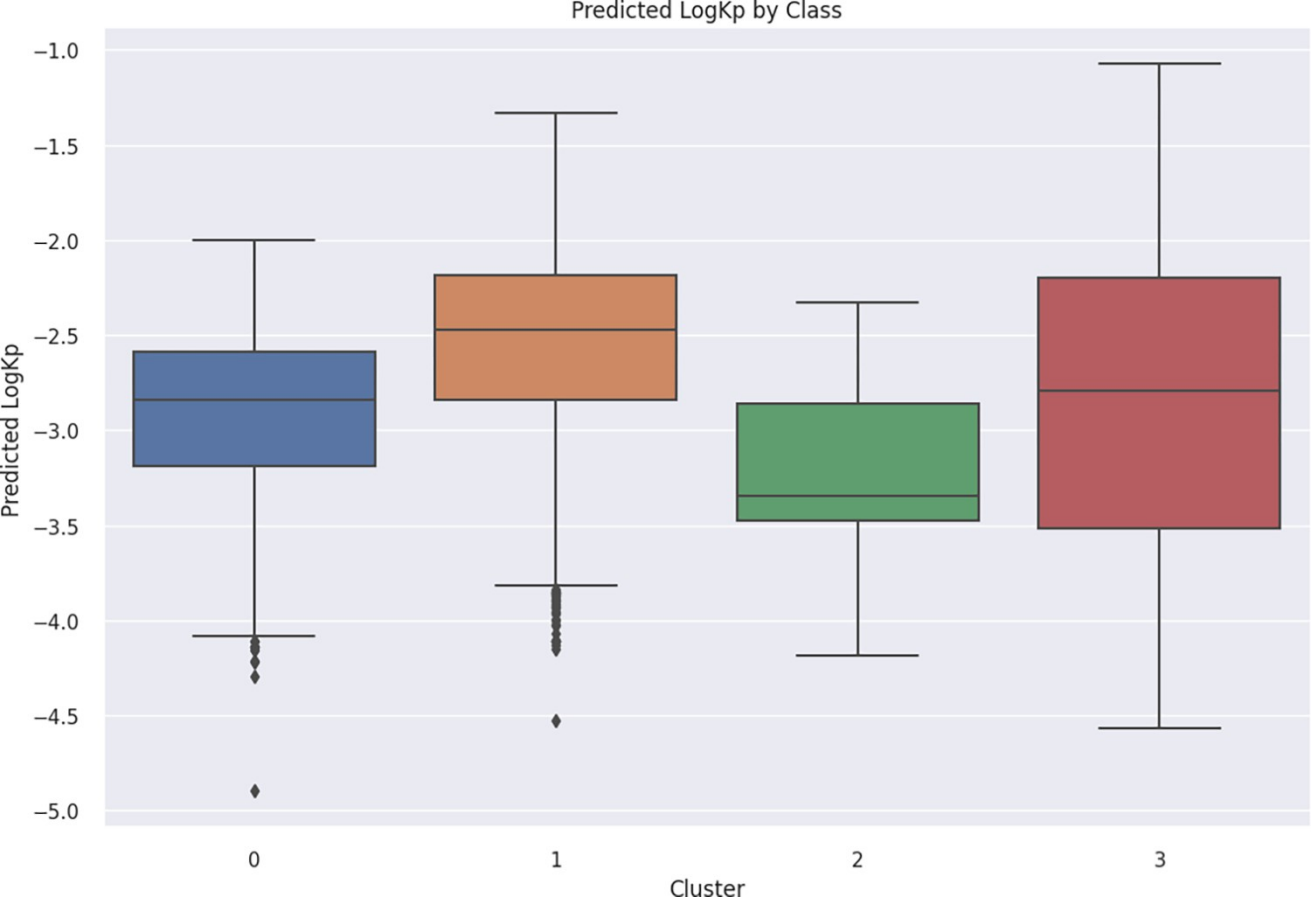
Predicted LogK_p_ Values for FDA-Approved Drug Clusters. Notably, pairwise comparisons indicated statistical significance between all clusters (*p* < 0.0001), except for the comparison between Cluster 0 and Cluster 3 (*p* = 0.029).

The LogK_p_ values of FDA-approved drugs were predicted using the LGBM model, and the corresponding results are presented in Fig 9. Among the clusters, Cluster 1 exhibited the highest predicted permeability (*p* < 0.0001), whereas Cluster 2 displayed the lowest permeability (*p* < 0.0001). Cluster 3 exhibited a wide range of permeability values.

### ATC groups permeability patterns

**Fig 10:**
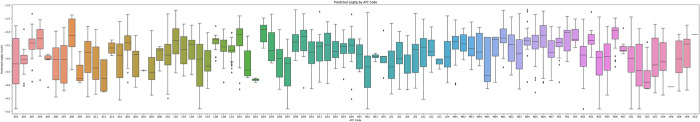
Box Plot of the Predicted LogK_p_ for FDA-Approved Drugs Categorized into 83 Classes Based on the First Two Levels of the ATC Code.

**Fig 11:**
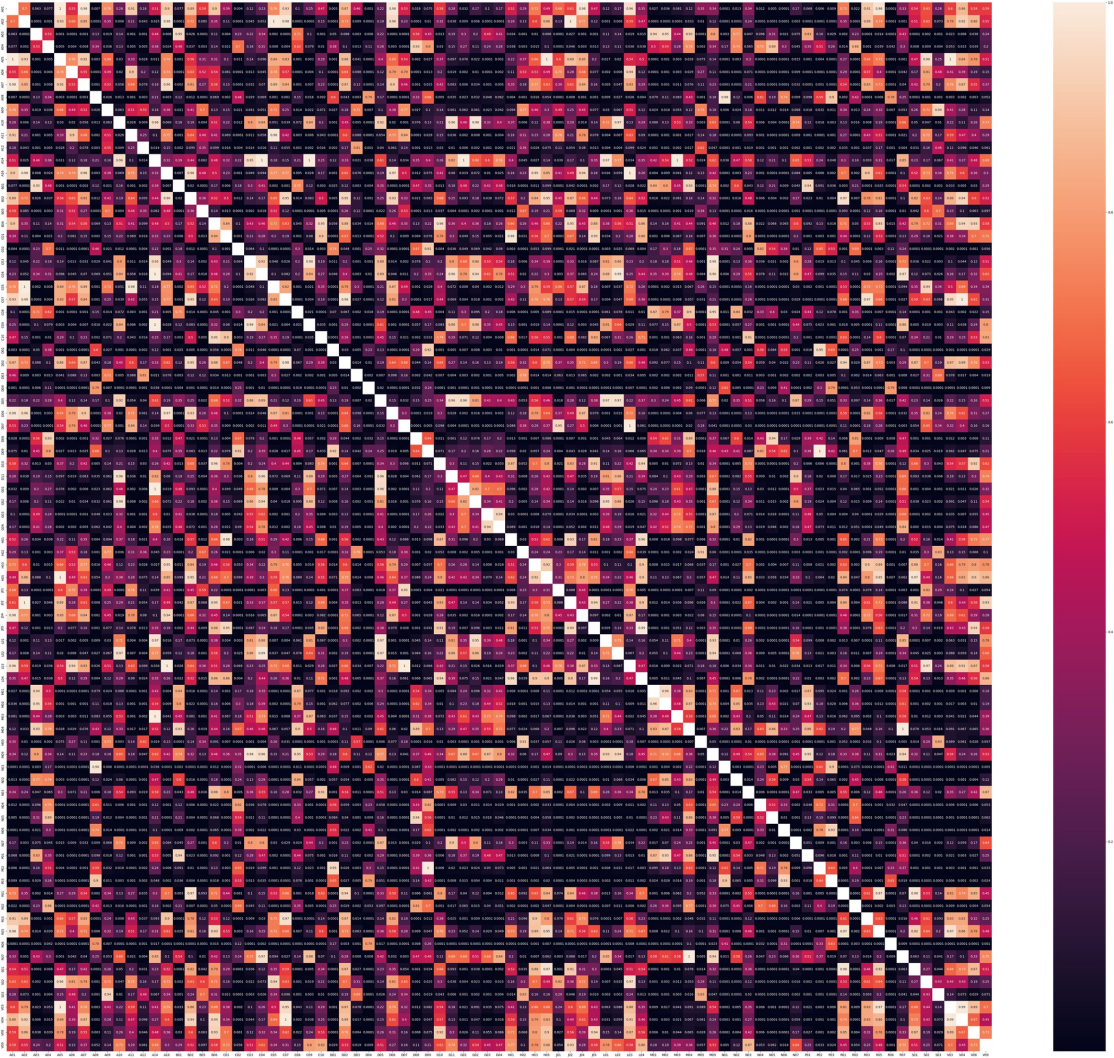
Statistical Comparison Heatmap for Predicted LogK_p_ Values Among ATC Drug Groups. The Mann-Whitney U test was employed for statistical validation, investigating distinct distributions of predicted permeability between groups. No control for multiple testing was applied due to the exploratory nature of the analysis, presenting these findings as hypotheses for further investigation.

A total of 2456 FDA-approved drugs were categorized into 83 classes based on the first two levels of the ATC code (Fig 10). The analysis revealed significant differences in the predicted LogK_p_ values among the different classes (*p* < 0.0001). Subsequent pairwise comparisons within the groups identified 1517 pairs with statistically significant differences (*p* < 0.05) out of a total of 3403 pairs examined, as shown in Fig 11. The statistical test was used to validate the different distributions of predicted permeability between groups. Notably, distinct LogK_p_ distributions were observed for certain groups. For instance, anesthetics (N01) which are composed of general anesthetics that are known to be highly lipophilic and readily pass the blood-brain barrier, and local anesthetics, which act by permeating skin layers. They exhibited significantly higher predicted LogK_p_ values than most of the other nervous system drug groups, including analgesics (N02) (*p* = 0.003), antiepileptics (N03) (*p* < 0.0001), psycholeptics (N05) (*p* = 0.005), and other nervous system drugs (N07) (*p* < 0.0001). Antihistamines for systemic use (R06) displayed significantly higher predicted LogK_p_ values (-2.09 ± 0.42 cm/h) compared to drugs for functional gastrointestinal disorders (A03) (*p* < 0.0001), which predominantly consisted of anticholinergic drugs. Furthermore, drugs for functional gastrointestinal disorders (A03), antiemetics (A04), and antiobesity drugs (A08) exhibited significantly higher predicted LogK_p_ values (-2.48 ± 0.51, -2.42 ± 0.60, and -2.17 ± 0.67 cm/h, respectively) compared to most other members of the alimentary tract and metabolism group (A). Within the cardiovascular system group, calcium channel blockers (C08) displayed significantly higher LogK_p_ values compared to beta blockers (C07) (*p* < 0.0001), while agents acting on the renin-angiotensin system (C09), including ACE inhibitors and ARBs, exhibited significantly higher LogK_p_ values than beta blockers (*p* = 0.004) but lower values than calcium channel blockers (*p* = 0.021).

## Discussion

In recent years, the transdermal route of drug administration has gained prominence as a convenient option for patients [4]. However, the skin presents a natural barrier that hinders the permeation of chemicals into the bloodstream. The ability of a drug to effectively penetrate the skin and reach the systemic circulation depends on its physicochemical properties. Therefore, the development of predictive models is crucial to identifying and screening potential drug candidates with higher bioavailability via the transdermal route. These models can assist in the selection of drugs that possess the necessary physicochemical characteristics for successful transdermal drug delivery. Previous QSPR studies have primarily focused on developing linear models using MLR and PCA [10]. These linear models offer a straightforward and easily interpretable approach for predicting skin permeability. However, it is important to note that linear models may oversimplify the relationship between physicochemical variables and compound descriptors when compared to non-linear models [15,27]. Our findings indicate that the performance of the MLR model was the poorest among the models evaluated in this study (R^2^ = 0.338, RMSE = 0.814, MAE = 0.624). In contrast, the performance of nonlinear machine learning models, particularly most of the boosting models such as LGBM, XGBoost, and Gradient Boosting, demonstrated superiority in predicting LogK_p_. Most boosting methods revealed higher performance over bagging models and ANNs. Moreover, ANN exhibited better performance than bagging models. These results are consistent with previous research findings [27].

The relationship between descriptors and skin permeability has been extensively studied in previous research [28,29]. In our investigation, we identified four descriptors, namely XLogP, number of hydrogen bond doners (nHBDon), number of hydrogen bond acceptors (nHBAcc), and topological polar surface area (TopoPSA), which exhibited a strong correlation with LogK_p_. Additionally, our analysis revealed that these descriptors ranked among the top features in the best-performing models, underscoring their relevance and contribution to accurate predictions of skin permeability. XLogP is a calculation method for LogP and represents a measure of molecular hydrophobicity [30]. LogP has been widely recognized for its significant association with skin permeability [29]. This observation is consistent with the understanding that hydrophobic molecules have an increased ability to traverse the hydrophobic barrier of the skin. Furthermore, we observed a highly negative correlation between LogK_p_ and the TopoPSA descriptor, which further emphasizes the role of molecule polarity in skin permeation. The number of hydrogen bond donors and acceptors in a molecule plays a significant role in its interaction with water molecules [31]. These functional groups can form strong hydrogen bonds with water, which require a considerable amount of energy to break. This interaction with water molecules can hinder the penetration of the molecule through the hydrophobic lipid layers of the skin. Interestingly, we also observed a negative correlation between the number of Lipinski failures and LogK_p_. Lipinski rules, originally developed to predict the oral bioavailability of drugs, are based on factors such as molecular weight, lipophilicity, the number of hydrogen bond donors, and the number of hydrogen bond acceptors [32]. The correlation between the number of Lipinski failures and skin permeability suggests the potential extension of these rules for predicting skin permeability [6].

FDA-approved drugs serve as a valuable resource for seeking suitable candidates for transdermal drug delivery systems. Despite the molecular diversity observed among these drugs, our analysis revealed their classification into four distinct clusters based on their descriptor properties. Cluster 0, comprising 301 drugs, exhibited high molecular weights, saturation, and polarity. Cluster 1, consisting of 980 drugs, exhibited lower molecular weights, high unsaturation, and aromaticity. Cluster 2, encompassing 61 drugs, mainly consisted of high molecular weight compounds, particularly polypeptides. Finally, Cluster 3 included 951 drugs with very low molecular weights. These clusters exhibited significant variation in their predicted LogK_p_ values, indicative of their distinct permeability characteristics. Notably, Cluster 1 exhibited significantly higher predicted LogK_p_ values compared to the other clusters (*p* < 0.0001). This may be attributed to the high degree of unsaturation and aromaticity within this group, coupled with its lower molecular weight. Conversely, Cluster 2 displayed significantly lower predicted LogK_p_ values compared to all other clusters (*p* < 0.0001). Although this cluster was not represented in the training dataset, it aligns with the general understanding that very high molecular weight compounds tend to have poor permeability. Additionally, the high number of hydrogen bond doners/acceptors contributes significantly to the poor bioavailability of drugs as it favors the interaction with water instead of membrane lipids [31]. Cluster 3 exhibited a wide range of predicted LogK_p_ values, reflecting the diverse nature of compounds within this cluster. The observed variability can be attributed to the broad spectrum of compounds classified under Cluster 3, each possessing unique physicochemical properties that impact their permeability.

ATC is an annotation system introduced by the World Health Organization that gives all drugs a code that classifies them based on five levels [26]. The first level represents the anatomical and pharmacologic group of the drug, where drugs are classified into one of the 14 groups. The second level represents the therapeutic and pharmacologic groups of the drug. This annotation system is used here to investigate the skin permeability patterns across the different pharmacologic and therapeutic groups of FDA-approved drugs. Several groups showed significant differences in predicted LogK_p_ values. For instance, anesthetics (N01) consist of general and local anesthetics. General anesthetics are characterized by high lipophilicity [33], enabling their rapid passage across the blood-brain barrier. Our findings suggest that the high skin permeability predicted for these compounds highlights their potential applicability in transdermal drug delivery. Substantiating our predictions, studies support our finding regarding fentanyl as a highly permeable drug with several transdermal patches on the market [34,35]. On the other hand, local anesthetics are relatively hydrophobic drugs that must permeate through the skin to act on dermis nerve terminals [36]. Hypertension is a chronic disease with an interest in increasing patient compliance using TDD [37]. Our results reveal higher predicted LogK_p_ for calcium channel blockers compared to beta blockers. Also, up to our knowledge, there were no studies that compared the permeability of antihypertensives from different pharmacologic groups. Our results are in agreement with previous comparisons with drugs from the same pharmacologic group, even though calcium channel blockers have no representation in our training dataset [38,39]. Our analysis provides a broad comparison of drug groups in terms of their permeability patterns. However, for a more detailed understanding of the permeability characteristics within individual pharmacological groups, the data are provided in S1 Data. This supplemental information allows interested readers to delve deeper into the specific permeability trends and explore the individual variations among different pharmacological categories.

The successful development of regression models for skin permeability prediction opens avenues for efficient drug design and discovery. The accurate prediction of LogK_p_ can aid in identifying molecules with optimal permeability characteristics, potentially reducing the need for time-consuming and costly experimental testing. The developed models have the potential to be valuable tools in early-stage drug discovery, development, and formulation processes. They can aid in the selection and optimization of compounds with desirable skin permeability properties, thereby facilitating the development of more effective and efficient drug candidates. The insights gained from the correlation analysis between descriptors and skin permeability can further inform the design and optimization of compounds with specific permeability profiles. Clustering analysis of FDA-approved drugs offers guidance in selecting and optimizing candidates for TDD and provides a unique perspective on skin permeability patterns across therapeutic groups. The study extends from earlier research and holds significant implications for drug development decisions, offering actionable insights for tailoring molecules, prioritizing candidates, and developing targeted and patient-friendly drug delivery systems [40,41]. Furthermore, guided by the principles of the 3Rs (replacement, reduction, and refinement) in animal research, there exists an ethical commitment to diminish the reliance on humans and animals in studies and to explore alternative methodologies, including *in vitro* and *in silico* models, for compound testing [42]. However, the ethical impact on industry and the need for guidelines to validate the predictions of these models, ensure transparency and interpretability, and prevent misuse of them highlight the importance of a balanced and accountable approach to harnessing the benefits of AI in drug development [43,44]. International organizations such as the OECD have developed guidance and recommendations for validating QSAR modeling, facilitating the regulatory acceptance and adoption of *in silico*-generated data, and ultimately aiming at minimizing the necessity for animals in toxicity studies [45]. Additionally, employing *in silico* QSAR modeling on data derived from human skin not only proves to be a cost-effective substitute for using animal skin but also yields predictions with enhanced accuracy in predicting human skin permeability.

The training set used in this study encompassed a broad range of molecules with diverse properties. However, it is important to acknowledge that the representation of high molecular weight drugs, such as proteins, was limited in the training set. Proteins, due to their unique characteristics and larger molecular sizes, may exhibit distinct behaviors and permeability patterns compared to small molecule drugs. In addition, it is important to acknowledge that not all drugs in the DrugBank database have assigned ATC codes, and some drugs, particularly proteins, may lack a defined SMILES structure. Consequently, the analysis based on ATC codes should be interpreted cautiously, considering the potential limitations and incomplete representation of certain drug classes, particularly proteins. Finally, no control for multiple testing was implemented, given the exploratory nature of this analysis, although we used group comparison using the Kruskal-Wallis test before each time multiple testing was conducted to validate the significance of the difference.

Future research should focus on integrating multi-omics data for a holistic understanding of skin permeability, the anatomical location of the skin, the impact of permeation enhancers, and the development of mechanistic models considering different mechanisms of drug permeability. Validation across diverse populations, exploration of novel descriptors, and improving machine learning model explainability are essential. Using cutting-edge models that are known to give outstanding performance without using descriptors can increase predictive accuracy and overcome the limited representation of the molecule [46]. However, the practical implementation of such models may pose challenges, requiring substantial computing resources, which may be limited. Economic impact studies are needed to evaluate the cost-effectiveness of AI-driven drug development.

## Conclusion

In conclusion, our study highlights the effectiveness of AI algorithms in predicting skin permeability for molecules. The boosting models, particularly LGBM, demonstrated superior performance in LogK_p_ prediction compared to other models, providing robust quantitative estimations. The comprehensive set of molecular descriptors and the optimization of model parameters contributed to the accuracy of the predictions with certain descriptors describing molecule polarity being highly correlated with the permeability. Furthermore, the cluster analysis of FDA-approved drugs uncovers distinct permeability profiles within different drug classes by revealing four clusters with distinct properties, facilitating the identification of potential drug candidates for TDD. Further classification by ATC code reveals drugs with promising TDD candidates within specific therapeutic categories. By integrating AI-based predictions of skin permeability, this research offers valuable insights and potential applications in early-stage drug discovery, formulation, and optimization, paving the way for efficient and targeted TDD systems with enhanced therapeutic efficacy.

## Supporting information

S1 Table Descriptive Statistics of Selected Molecular Descriptors for Identified Clusters. Note that the table presents the average ± standard deviation of key molecular descriptors characterizing each cluster derived from the DrugBank dataset of FDA-approved drugs.
(DOCX)

S1 Data The LGBM predicted LogK_p_ for the FDA-approved drugs along with their ATC code and cluster. (XLSX)
