# Supplementary material for: Predictive modeling of skin permeability for molecules: Investigating FDA-approved drug permeability with various AI algorithms
Source: PLOS Digit Health. 2024 Apr 3;3(4):e0000483. doi: 10.1371/journal.pdig.0000483 (PMC10990209; doi:10.1371/journal.pdig.0000483)
Supplement: S1 Table — Note that the table presents the average ± standard deviation of key molecular descriptors characterizing each cluster derived from the DrugBank dataset of FDA-approved drugs. (DOCX) [file pdig.0000483.s001.docx]

**Supplementary Data**

**S1 Table.** **Descriptive Statistics of Selected Molecular Descriptors for Identified Clusters.** Note that the table presents the average ± standard deviation of key molecular descriptors characterizing each cluster derived from the DrugBank dataset of FDA-approved drugs.

| **descriptors** | **Class 0** | **Class 1** | **Class 2** | **Class 3** |
| --- | --- | --- | --- | --- |
| **MW** | 540.98 ± 193.56 | 423.85 ± 116.12 | 1529.25 ± 605.62 | 231.17 ± 78.60 |
| **nHBDon** | 2.68 ± 2.70 | 1.82 ± 1.86 | 18.30 ± 10.13 | 1.83 ± 1.54 |
| **nHBAcc** | 7.21 ± 4.88 | 5.55 ± 3.33 | 34.00 ± 16.68 | 3.77 ± 2.32 |
| **TopoPSA** | 119.43 ± 76.24 | 93.06 ± 60.58 | 581.51 ± 293.54 | 70.51 ± 43.72 |
| **LipinskiFailures** | 1.25 ± 1.28 | 0.64 ± 0.99 | 4.10 ± 0.54 | 0.17 ± 0.50 |
| **HybRatio** | 0.70 ± 0.16 | 0.38 ± 0.19 | 0.55 ± 0.15 | 0.48 ± 0.31 |
| **khs.aaCH** | 2.04 ± 3.48 | 6.37 ± 3.53 | 10.66 ± 7.36 | 2.70 ± 2.71 |
| **naAromAtom** | 4.71 ± 7.25 | 12.09 ± 5.53 | 18.87 ± 13.01 | 4.58 ± 4.19 |
| **bpol** | 54.18 ± 21.17 | 36.21 ± 12.37 | 135.93 ± 60.10 | 21.29 ± 10.08 |

The dataset underwent PCA, leading to the identification of four distinct clusters (Classes 0 to 3) with varying properties. The descriptors include molecular weight (MW), number of hydrogen bond donors (nHBDon), number of hydrogen bond acceptors (nHBAcc), topological polar surface area (TopoPSA), Lipinski rule failures, hybridization ratio (HybRatio), number of aromatic carbons (khs.aaCH), number of aromatic atoms (naAromAtom), and total absolute sum of polarizability difference between bonded atoms (bpol). These descriptors provide insights into the chemical composition and diversity within each cluster, offering a basis for understanding the variability in skin permeability across FDA-approved drug compounds.
